# The Backup That Led to a Blackout: Syncope and Severe Constipation

**DOI:** 10.7759/cureus.72802

**Published:** 2024-10-31

**Authors:** Anupa Ghimire, Sherell Hicks

**Affiliations:** 1 Emergency Medicine, University of Alabama at Birmingham Heersink School of Medicine, Birmingham, USA

**Keywords:** compression of ivc, fecaloma, severe constipation, stercoral proctocolitis, syncope

## Abstract

Syncope is a common presentation in the emergency department (ED); however, it is rarely due to stercoral proctocolitis causing inferior vena cava (IVC) compression. We describe the case of a young male patient who presented to the ED after having a syncopal episode. Through obtaining a detailed history and physical exam, he was found to have abdominal distention and endorsed constipation. He was diagnosed with severe fecal impaction causing stercoral proctocolitis and IVC compression causing intermittent syncope. He underwent an emergent exam under anesthesia and fecal disimpaction in the operating room. This case emphasizes the importance of doing a thorough physical exam to avoid missing a complication of this rare diagnosis.

## Introduction

Syncope, defined as a transient loss of consciousness due to global cerebral hypoperfusion, accounts for 3-6% of all emergency department (ED) visits annually [1,2]. The suspected etiology dictates management as the causes of syncope range from benign conditions (i.e. vasovagal response) to treatable causes (i.e. dehydration, medication effects, anemia) to life-threatening conditions (i.e. arrhythmias, myocardial infarction, intracranial abnormalities). Vasovagal syncope is the most frequent cause, followed by cardiac causes and orthostatic hypotension. Despite thorough evaluation, a significant number of cases remain unexplained [3,4].

The diagnostic approach to syncope involves a systematic assessment, starting with a thorough history and physical examination, which can lead to a diagnosis in approximately 50-65% of cases [2,5,6]. Important considerations include the patient’s medical history, the context of the syncopal episode, and associated symptoms such as the presence or lack of prodromal symptoms, chest pain, or dyspnea. This case highlights the value of spending time at the bedside performing a thorough history, review of systems, and physical exam in order to recognize and appropriately manage abnormal findings as patients may be unaware of how their acute presentation is linked to a chronic issue. 

## Case presentation

We present a case of a 26-year-old male patient with no reported significant past medical history who presented to the ED after an episode of syncope. The patient was at the hospital visiting family when he suddenly felt lightheaded and flushed. He lowered himself to the ground and lost consciousness. He had a similar episode of syncope while straining for bowel movement a week earlier but did not seek medical care at that time. He is not currently on any medication. He denied palpitations, preceding chest pain, or shortness of breath. He reported being asymptomatic during our initial evaluation. During review of systems, he endorsed constipation, reporting that he had not had a normal bowel movement in almost two months. He was passing flatus but required a lot of straining. He denied any nausea, vomiting, rectal bleeding, hematochezia, or significant abdominal pain. Upon further questioning, he reported having issues with constipation since childhood. He trialed polyethylene glycol in the past as recommended by his primary care provider with minimal relief. He was referred to a gastroenterologist but never made an appointment. He denied any surgeries in the past. He denied using any illicit drugs including opiates. He occasionally drinks alcohol and smokes cigarettes and marijuana daily. 

His initial vital signs were within normal limits with a heart rate of 88 beats per minute (bpm), a respiratory rate of 17 breaths per minute, a blood pressure of 118/85 millimeters of mercury, and oxygen saturation at 95% on room air, and he was afebrile at 98.5 degrees Fahrenheit. His physical exam was remarkable for a distended and firm abdomen without rigidity and minimal generalized tenderness to palpation. Bowel sounds were present. No surgical scars, hernia, or obvious mass was noted. An electrocardiogram upon arrival showed sinus rhythm at 80 bpm with no signs of arrhythmia or ischemic changes. 

**Figure 1:**
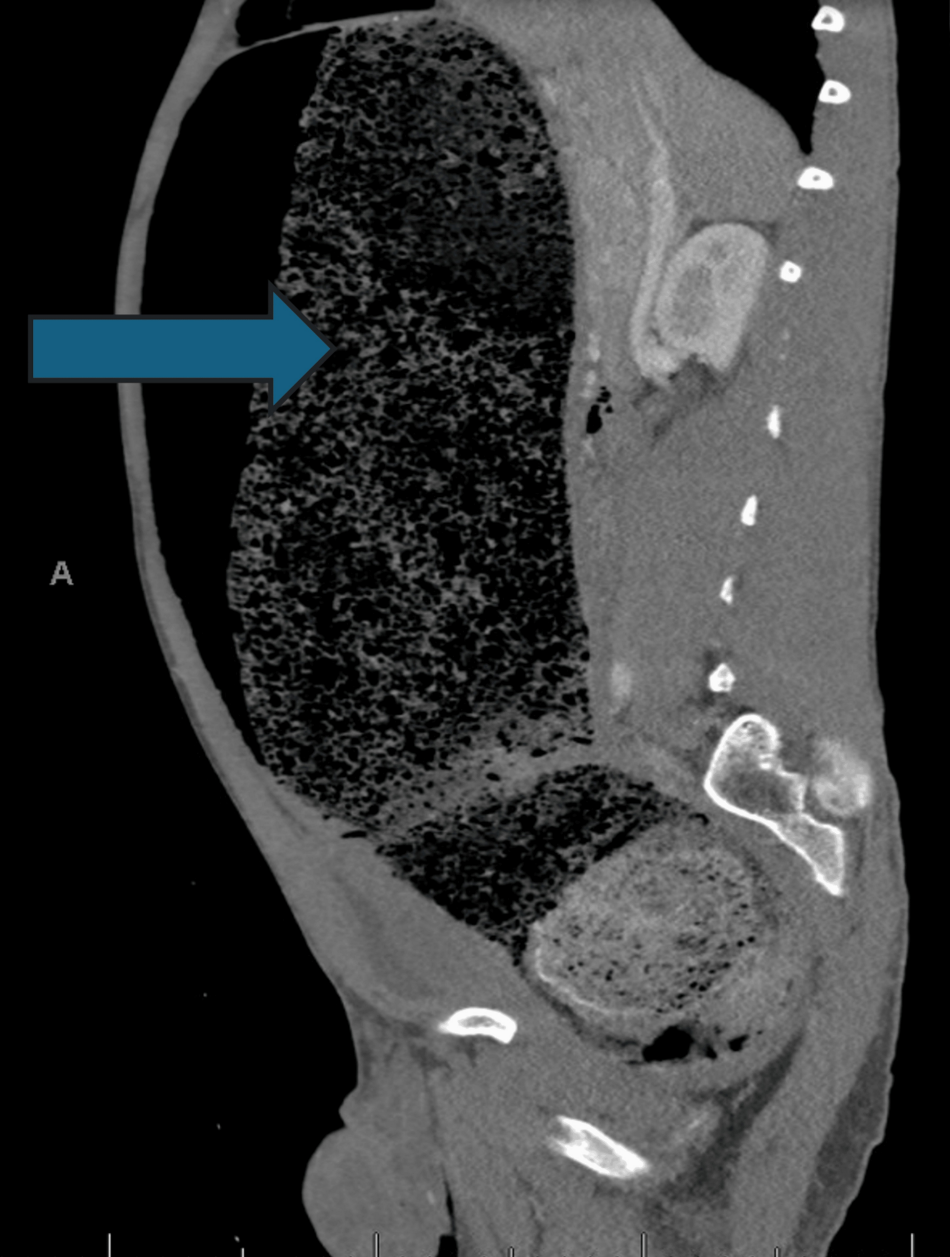
CT sagittal view of abdomen/pelvis showing gigantic fecaloma (blue arrow) with compression of abdominal structures

**Figure 2:**
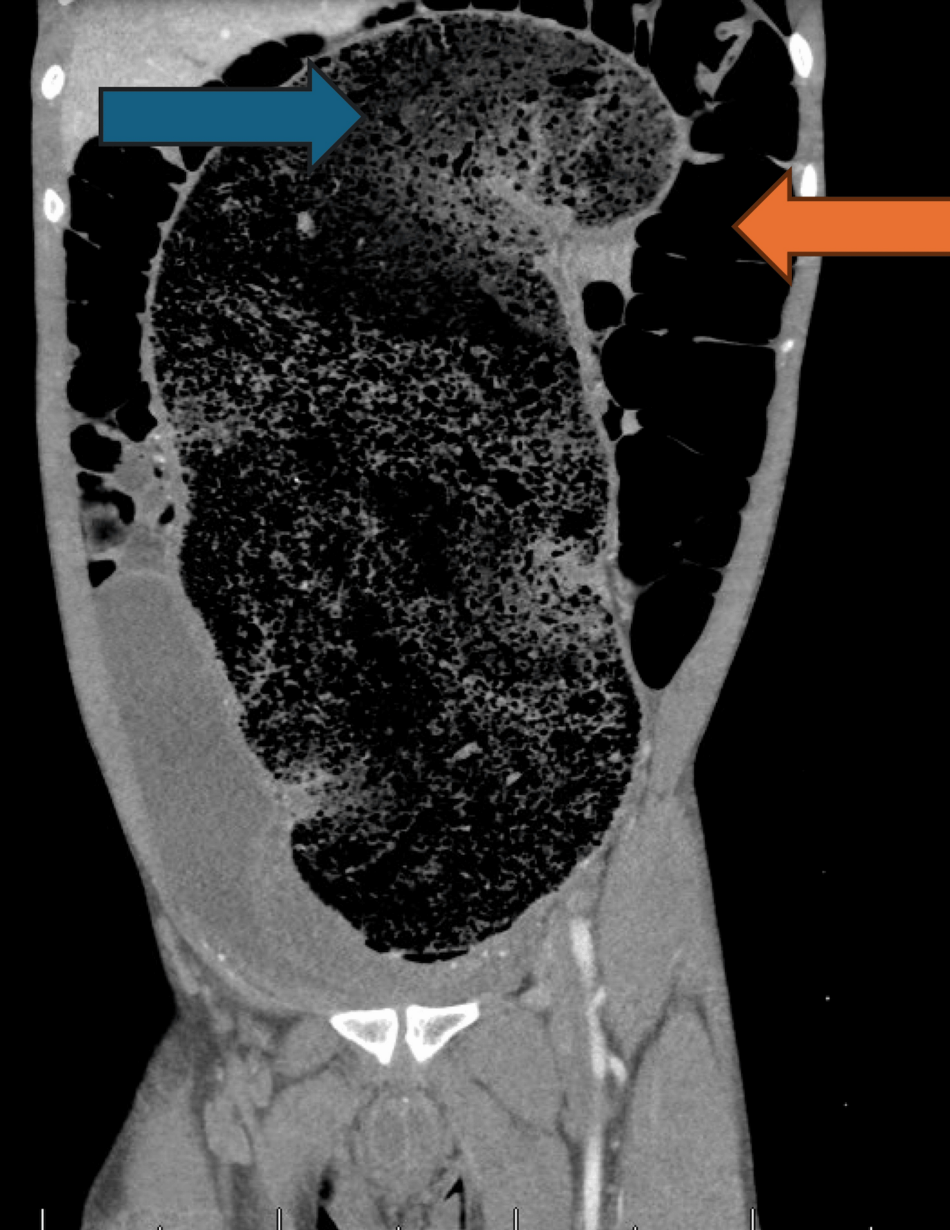
CT coronal view with gigantic fecaloma (blue arrow) and dilated loops of bowel (orange arrow)

**Figure 3:**
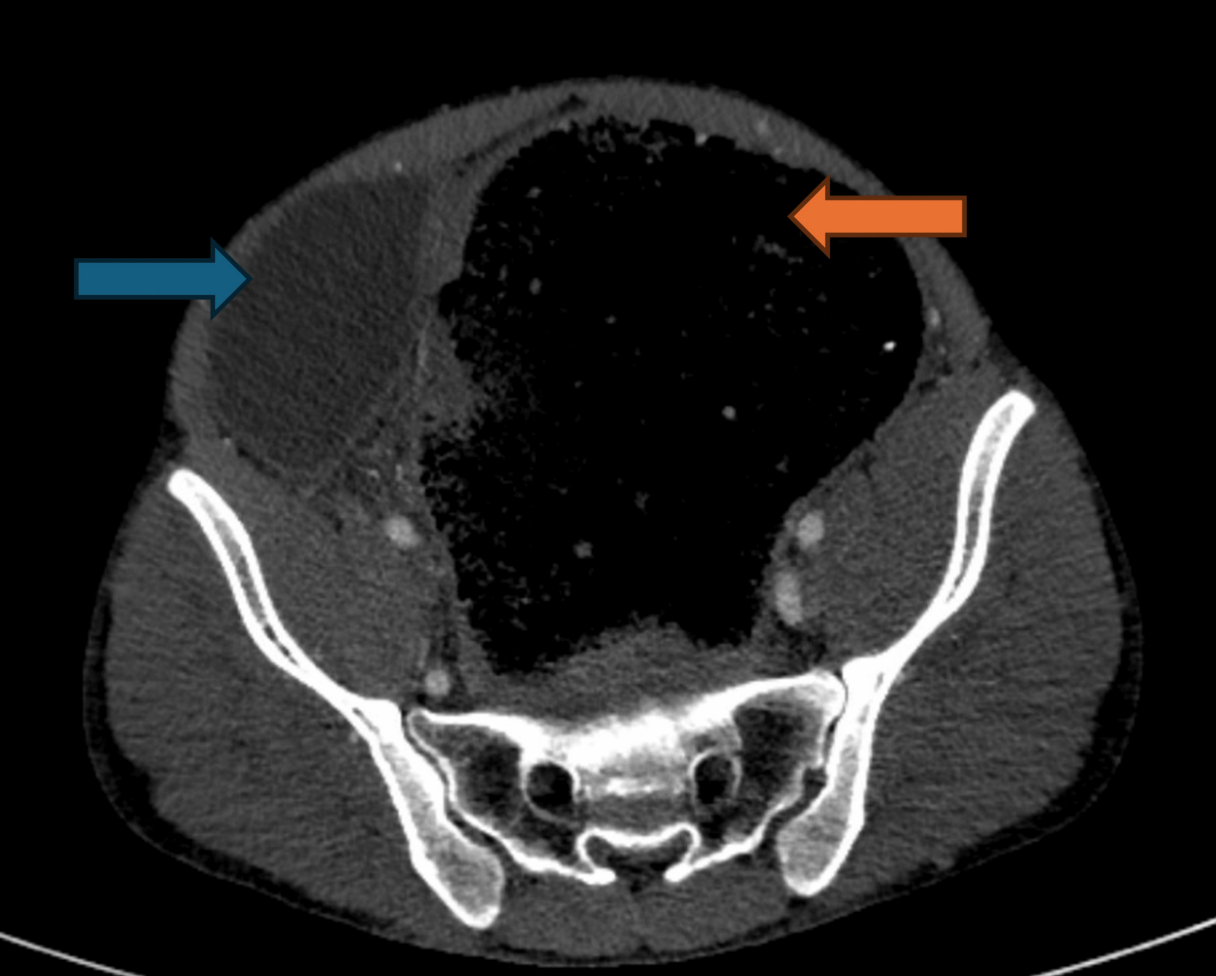
CT axial view of pelvis showing large intestinal gas burden (orange arrow) with displacement of the urinary bladder to the right (blue arrow)

Labs were unremarkable without leukocytosis, acute anemia, or electrolyte derangements. Given the significant abdominal distention in an otherwise healthy, young patient, a computed tomography (CT) scan of the abdomen and pelvis was obtained for further evaluation, which showed severe rectal and colonic fecal burden with gigantic fecaloma producing a mass effect on central abdominal structures and compressing the inferior vena cava (IVC) (Figure 1). The transverse colon was dilated to 15cm (Figure 2). The fecaloma and intestinal gas burden caused significant displacement of the urinary bladder to the right (Figure 3). There were associated colonic and rectal wall thickening. These findings were consistent with stercoral proctocolitis without evidence of perforation or signs of peritonitis. 

Surgery was consulted for evaluation, who attempted decompression with a red rubber catheter at bedside. This maneuver resulted in a significant rush of air without stool output; therefore, the patient was taken for exam under anesthesia and manual fecal disimpaction in the operating room (OR) given the severe fecal impaction with colonic distention. A massive amount of stool in the descending colon was disimpacted manually and with irrigation in the OR. After the surgery, his abdomen was significantly decompressed. The patient tolerated the procedure well and remained hemodynamically stable after the procedure. The abdominal X-ray post procedure showed a decrease in stool burden, and the patient showed significant clinical improvement. Gastroenterology was consulted for further evaluation of his constipation. Thyroid function tests and the IgA level were ordered, both of which were within normal limits. The patient was treated with polyethylene glycol, sennosides, and tap water enemas every 48 hours. He was discharged with instructions for dietary modification as well as prescriptions for senna and polyethylene glycol. He was advised to follow up closely outpatient with gastroenterology to monitor his chronic constipation to prevent developing recurrent complications.

## Discussion

This young patient had no acute abdominal complaints upon presenting to the ED for syncope. While obtaining a thorough history and exam, the patient’s abdominal distention led to questioning which revealed chronic constipation and led to further workup.

Vasovagal syncope is a common cause of syncope and can be associated with straining during defecation; however, our case is unique in that syncope occurred due to the significant stool burden from chronic constipation compressing the IVC. The increased fecal burden caused mechanical compression of the IVC, decreasing preload and cardiac output resulting in decreased cerebral perfusion, leading to syncope. Chronic constipation, particularly when left untreated, can lead to significant complications, including stercoral proctocolitis, a rare condition where fecalomas cause inflammation, ulceration, and impingement on nearby structures [7].

Stercoral proctocolitis is a rare complication of chronic constipation and fecal impaction characterized by ischemic and inflammatory injury to the colonic wall secondary to prolonged fecal impaction [8]. The injury to the gut wall comes from the development of fecalomas, which is a mass of dehydrated fecal material. Its radiologic findings also include signs of gut wall inflammation like thickening of the colonic wall and stranding of peri-colonic fat [9]. A fecaloma that increases significantly in size can have a mass effect on surrounding structures, which can cause secondary symptoms including syncope, as in this case. About 27% of patients develop multiple areas of focal ulceration [10]. Without treatment, stercoral proctocolitis can progress to transmural inflammation, ulceration, and in severe cases, perforation, which carries a mortality rate of up to 60% [11]. Management requires prompt decompression via bowel regimen or disimpaction in the OR, as delay can lead to perforation, peritonitis, and septic shock, significantly increasing morbidity and mortality [12-14]. To reduce the risk of developing this rare complication, patients with constipation should follow a bowel regimen that includes laxatives and stool softeners, along with dietary modifications to prevent excessive stool accumulation.

When determining management for a patient who presents with syncope, workup is dependent on maintaining a broad differential given the diverse etiologies. Although rare, this case shows how a comprehensive history and exam led to this diagnosis. Abdominal imaging is not recommended as a routine part of a syncopal workup; however, this case highlights the importance of relying on your exam to gain insight into how a chronic disease process like constipation can cause an acute ED presentation like syncope. 

## Conclusions

This case describes a rare cause of syncope. A comprehensive history and physical exam led to the diagnosis of constipation causing the rare complication of stercoral proctocolitis with IVC compression resulting in syncope. Keeping a broad differential and relying on the history and abnormal exam findings like constipation and abdominal distention can clue medical providers into making the atypical but correct diagnosis.
